# Effects of the Addition of *Trichoderma reesei* Cellulase to Broiler Chicken Diets for a 21-Day Period

**DOI:** 10.3390/ani14101467

**Published:** 2024-05-15

**Authors:** Francisco dos Santos Perim, Weslane Justina da Silva, Dênia Oliveira de Souza, Cirano José Ulhoa, Camila Ferreira Rezende, Ludmilla Faria dos Santos, Fabiana Ramos dos Santos, Fabiano Guimarães Silva, Cibele Silva Minafra

**Affiliations:** 1 Goiano Federal Institute of Education Science and Technology (Instituto Federal Goiano—IF Goiano), Rio Verde 75901970, Brazil; francisco.perim@cobbgenetics.com (F.d.S.P.); deniaarcanjo@gmail.com (D.O.d.S.); camilagyn_88@hotmail.com (C.F.R.); vetludmilla@gmail.com (L.F.d.S.); fabiana.santos@ifgoiano.edu.br (F.R.d.S.); fabiano.silva@ifgoiano.edu.br (F.G.S.); 2 Program Postgraduate in Biotechnology and Biodiversity of Goiás—PGBB/UFG, Federal University of Goiás, Samambaia Campus, Goiânia 74001240, Brazil; weslanejds@gmail.com; 3 Department of Biochemistry and Molecular Biology, Biological Sciences Institute, Federal University of Goiás, Samambaia Campus, Goiânia 74001240, Brazil; ulhoa@ufg.br

**Keywords:** aviculture, enzyme, metabolism, organ biometrics, performance

## Abstract

**Simple Summary:**

The use of enzymes in the feed of chickens and the production animals is already well established, given the nutritional and economic benefits that can be achieved without affecting the quality of the final product. Among research efforts, new discoveries are made every day regarding the potential of products developed to provide efficient enzymes as additives in animal diets. Therefore, this study investigated the influence of enzymes produced from the cultivation of *Trichoderma reesei* in powder and liquid forms on the feeding of broiler chickens from 1 to 21 days of age. The diets were composed of corn and soybean meal, and this study evaluated their effects on performance, digestibility, blood parameters, and the biometry of the digestive system. The enzymes produced, cellulases, affected feed intake, digestibility, and blood electrolyte concentrations were quantified. However, the biometry of the digestive system and analyses of pancreas and liver viscera were not altered, which may indicate normal bird development and the absence of toxicity. In conclusion, *Trichoderma reesei* cellulases are an interesting option for use in animal diets.

**Abstract:**

The cellulose present in the cell wall of vegetables prevents the greater release of nutrients to the animal. Therefore, the use of the cellulase enzyme is a viable strategy as it is capable of breaking cellulose bonds, releasing nutrients such as glucose, increasing dietary energy, and thus improving the productive performance of birds. *Trichoderma reesei* is efficient in the production of cellulase, which is produced via submerged fermentation followed by purification, formulation, and drying. Therefore, an experiment was carried out using 240 male broilers of the Cobb-500^®^ lineage to verify the effects resulting from the addition of powdered (500 g/t and 1000 g/t) and liquid (500 mL/t) cellulase over a period of 1 to 21 days. A completely randomized experimental design was used, consisting of four treatments with six replications and ten birds per replication that were housed in an experimental cage. It was observed that performance and digestibility results were significantly different with cellulase supplementation. Also, the relative weight of the large intestine in the period between one and seven days increased when cellulase was added at 1000 g/t. In the period of between eight and 14 days of life, the birds that consumed only the basal diet obtained higher levels of liver protein than those that received the treatments with the addition of the enzyme. However, 15 and 21 days, the consumed feed effect did not occur between thus, it is not conclusive whether hepatotoxicity occurs with the addition of cellulase. For the blood parameters, at 21 days, the diets with added cellulase were not significantly different regarding electrolytes. It was concluded that this cellulase produced by *Trichoderma reesei* can be included in the animals’ diet.

## 1. Introduction

The study of enzymes has been of interest to several researchers, as these additives are incorporated into animal feed with the purpose of improving the utilization of scarce nutrients, providing better performance of the birds, and thereby increasing productivity [1]. Due to fluctuations in the prices of ingredients used in animal feed, enzymes play an important role in optimizing the use of nutrients in a more profitable way with a low environmental impact [2], being an additive that impacts animal production and contributes to the quality of products offered by broiler chickens, meat, and commercial cuts appreciated by consumers [2,3,4].

Due to the presence of structural polysaccharides in the cell walls of grains used as poultry feed, which hinder the nutritional utilization of the feed, the use of exogenous enzymes obtained from microrganisms such as fungi improves grain degradation, releasing a greater supply of nutrients for the animals. In addition to the nutritional benefits, enzymes also significantly influence the economy of the farmer who uses them in the animals’ diet [5,6,7].

Enzymes that degrade fiber are added to feed and thus improve bird nutrition, with the main effect of reducing the antinutritive factor of cereal fibers. Thus, enzymes improve digestion and the availability of nutrients for absorption in the intestine, improving animal growth [8]. Non-starch polysaccharide (NSP) enzymes are used in poultry diets to break down the NSPs found in plant cell walls, mainly pentoses, arabinoxylans, and β-glucans, thus releasing the nutrients present in the grain cell wall. This results in improved performance in the viscosity of the digesta and increased absorption of essential elements for the metabolization of chicken meat, carbohydrates, proteins, lipids, vitamins, and minerals [9,10].

Among enzymes, cellulase is an economically important enzyme, and it is sold on a large scale for various processes such as for use in animal feed. Cellulases belong to the glycosyl hydrolase family and are classified as endo β, 1-4 glucanases (EC 3.2.1.4); exo β, 1-4 glucanases (EC 3.2.1.176), also known as cellobiohydrolases (CBH); and β-glucosidases (EC 3.2.1.21) according to the IUBMB (International Union of Biochemistry and Molecular Biology). Therefore, the enzymatic complex affects the various components of cellulose, and through its combined activity, cellulose is hydrolyzed into glucose [11].

The objective of this study is to evaluate the addition of cellulases produced by *Trichoderma reesei* in powder and liquid form to the feed of broiler chickens from 1 to 21 days, in the diets composed of corn and soybean meal, where the effect of the diet on the performance, digestibility, blood parameters, and biometry of the digestive system were analyzed.

## 2. Materials and Methods

### 2.1. Broiler Farming

The experimental breeding of broiler chickens was carried out in the aviary of the Instituto Federal Goiano (Instituto Federal Goiano—Campus Rio Verde), in the Animal Nutrition and Biochemistry and Animal Metabolism laboratories, and in the Enzymology department of the Institute of Biological Sciences of the University Federal District of Goiás in the municipality of Goiânia, state of Goiás (GO), Brazil.

For the study, 240 male Cobb-500^®^ (Agua Clara, MS, Brazil) broilers were used, starting with birds with an average weight of 43.00 ± 0.82 g (average weight on the first day). The chicks were housed in 24 cages measuring 0.9 × 6 m each, equipped with pendulum drinkers and tubular feeders, and the heating source used was 100 watt light bulbs. From the first to the seventh day, the lamps remained lit for 24 h a day, and from the 8th to the 21st day, the lamps were lit from 9 am to 6 pm. The average maximum and minimum temperatures were 30.9 and 24.6 °C, respectively, while the average maximum and minimum humidity values were 48 and 43%, respectively, measured daily using a thermohydrometer.

The animals were distributed in a completely randomized experimental design, with four treatments, six replications and ten birds per experimental cage. The treatments were as follows: basal diet, basal diet with 500 g/t of cellulase powder, basal diet with 1000 g/t of cellulase powder, and basal diet with 500 mL/t of liquid cellulase.

The pre-starter ration was provided from the first to the seventh day, and the starter ration was provided from the eighth to the twenty-first day. The diets were formulated following the recommendations of the Brazilian tables for poultry and swine [12], as shown in Table 1. Water and feed were provided to the birds ad libitum throughout the experimental period.

### 2.2. Origin of the Tested Enzyme

The cellulases were produced by Trichoderma reesei via submerged fermentation. The powdered cellulase, with a FPase activity of 35.245 U/mL, is stable at temperatures of 40–80 °C and pH 3.0–7.5, with an optimal temperature of 65–75 °C and optimal pH of 4.5–6.0 (500 g/t and 1000 g/t). Liquid cellulase, with a FPase activity of 27.4 U/mL, is stable at temperatures of 40–80 °C and pH 3.0–7.5, with an optimal temperature of 65–75 °C and optimal pH of 5.5–7.0 (500 mL/t).

### 2.3. Performance Evaluation

At 1, 7, 14, and 21 days of age, the average weight of the chickens in each pen was evaluated to verify their weight gain, feed consumption, and feed conversion index during the observed period.

### 2.4. Apparent Metabolizability Analysis

To check the metabolization of the nutrients, the excreta produced by the birds were collected between the 4th and 7th day, and 14 to 17 days, twice a day. After collection, the excreta and diets were identified, froze, and subsequently sent to the Animal Nutrition Laboratory at IFGoiano, Campus Rio Verde, where the dry matter (DM) and crude protein (CP) contents were measured [13].

Of the excreta samples used for analysis, portions were collected, identified, pre-dried in a straight oven with forced ventilation (FANEM LTDA) at 55 ± 5 °C, and crushed in Wiley-type mills [13]. According to the experimental diets, The dry matter of the excreta (feces) was obtained after being dried in an oven with forced ventilation at temperatures of 55 °C ± 5 °C for 72 h. Analyses of the dry matter of the experimental feed and excreta in an oven regulated at 105 °C for 12 h were carried out to obtain the total nitrogen in the experimental feed and excreta using the micro-Kjeldahl method. Subsequently, the crude protein values were calculated by multiplying the %N by 6.25, resulting in the difference between the ingested and excreted nutrients divided by the nutrient. The dry matter retention value was obtained based on the amount of dry matter ingested, which was subtracted from the amount excreted in relation to weight gain. The crude protein retention was measured based on the amount of crude protein ingested, which was subtracted from the amount excreted divided by the animal’s weight gain [13]. The calculation of nutrient retention refers to the nutrient balance and weight gain, which were recorded in the periods of 4 to 7 and 14 to 17 days.

### 2.5. Intestinal Histomorphometry

One bird per cage was identified and transported to the slaughterhouse at the IF GOIANO aviary, Campus Rio Verde, and slaughtered via cervical dislocation. The procedure was conducted at 7, 14 and 21 days post-hatch. The birds were eviscerated, and the liver, gizzard, proventriculus, and pancreas, comprising the gastrointestinal tract, were collected (GIT). Then, the length of the GIT was determined. The proventriculus and the gizzard, pancreas, small intestine, large intestine, and liver (without the gallbladder) were then measured and weighed. All of the observed weights were recorded in Excel tables and used to calculate the relative weight of each animal.

The liver and pancreas were removed. After weighing, they were identified, stored, and quickly frozen using liquid nitrogen to interrupt enzymatic activity. In the analysis, 1 g of crushed tissue was collected and homogenized with 9 mL of water and then centrifuged at 8000 rpm at 40 °C for 10 min. The collected supernatant allowed for the determination of the amylase levels in the pancreas and the protein content and enzymatic activity of alkaline phosphatase and transaminases in the liver. These experiments were carried out in triplicate using commercial kits (Doles^®^, Goiás, Brazil). The procedures were carried out in an ice bath with distilled water to avoid the loss of enzyme activity.

### 2.6. Serum, Liver, and Pancreas Biochemical Profiles

The blood of the birds slaughtered via cervical dislocation was collected (7, 14, and 21 days old) via cardiac puncture and placed in labelled tubes. The tubes were then centrifuged at 6000 rpm for 10 min to obtain the serum. The serum was used for colorimetric determination of calcium (Ca) (mg/dL), phosphorus (P) (mmol/L), chlorine (Cl) (mmol/L), and potassium (K) mmol/L), and to determine alkaline phosphatase (AP) (IU/L) and protein (Prot) (g/dL) activity using commercial kits (Dolles^®^, Goiás, Brazil).

### 2.7. Statistical Analysis

Statistical analyses of the data were performed using a one-way ANOVA using Statistical and Genetic Analysis System (Saeg, UFV, Viçosa, Brazil), version 9.5. The normality distribution of the data in the treatments was confirmed using a Shapiro–Wilk’s test. The homogeneity of variance was determined using Levene’s test. The means were compared using Tukey’s test at *p* < 0.05 and *p* < 0.01 [14].

## 3. Results

Table 2 shows the ration consumption, weight gain, and feed conversion in the pre-starter and starter stages of broilers chickens fed cellulase-supplemented rations. No influence of cellulase enzyme supplementation on the weight gain and feed conversion parameters was observed, and the addition of powdered cellulase at 1000 g/t significantly improved feed intake during this period compared to the other treatments.

There was a significant difference in the weight gain and feed conversion between the groups. Providing powdered cellulase at 500 g/t resulted in better feed conversion than the other treatments, as well as significantly better weight gain compared to the addition of liquid cellulase.

Table 3 presents the digestibility data for the collection period from 4 to 7 and 14 to 17 days.

In the period of three to seven days, there was a significant effect the on nitrogen consumption in the dry matter, the nitrogen excreted in the dry matter, and nitrogen balance and retention. These results were verified based on the metabolic evaluation between 14 and 17 days. There was a statistical difference in the nitrogen consumption in matter drought, nitrogen balance, and nitrogen retention.

At eight days, when powdered cellulase at 500 g/t was added, the results showed a significant improvement in the nitrogen consumption in the dry matter, a better balance, and consequently better nitrogen retention. The nitrogen excretion levels were significantly lower than in the other treatment groups when powdered cellulase at 1000 g/t was added compared to the other treatments. The basal diet treatment had the highest nitrogen excretion in the dry matter and had a lower nitrogen retention compared to the addition of powdered cellulase (500 g/t) and liquid cellulase (500 mL/t).

The results obtained during the evaluated phases regarding the relative biometry for the pre-initial and initial phases are presented in Table 4.

There was no significant difference in the relative weight of the liver, proventriculus + gizzard, gastrointestinal tract, pancreas, small intestine, or gastrointestinal tract length during the three evaluated phases. The relative weight of the large intestine from day one to day seven was significantly lower when cellulase was not added, regardless of the type or concentration [15]. This greater development was attributed the probably due to the early provision of food and water, as likely occurred in this experiment.

The means of the pancreas weights throughout the experimental period are presented in Table 5. There was no significant difference in the absolute weight of the pancreas in any of the evaluated phases. 

The means of pancreatic amylase activity from one to seven days, eight to fourteen days, and fifteen to twenty-one days are presented in Table 6. For both types and concentrations of cellulase used for both phases, there was no significant difference in the pancreatic amylase activity.

For both types and concentrations of cellulase used for both phases, there was no significant difference in the pancreatic amylase activity. The average liver weights are found in Table 7. Throughout the experimental period, there was no significant difference in the absolute weight of the liver with the addition of the cellulase enzyme. The results showed a gradual increase in the liver weight as the birds’ age increased. This refers to the normal growth of the visceral organs in fast-growing birds.

The tissue enzymatic parameters of alkaline phosphatase (FA), glutamate–oxaloacetate transaminase (GOT), glutamate–pyruvate transaminase (GPT), and liver protein levels are found in the Table 8.

In this study, there were alterations in protein levels and GPT only in the period between 8 and 14 days when cellulase was added to the feed. The birds consuming only the basal diet obtained a higher percentage of protein than the birds receiving the other treatments, as well as lower GPT levels with the addition of cellulase at 1000 g/t and 500 mL/t. These parameters remained unchanged in the 15 to 21-day period. This may be explained by some metabolic alterations in the birds that could have influenced these values, such as heat or cold stress, absent or low water consumption, food restriction, or even bird handling for material collection.

There was no significant difference between the treatments in the periods between one and seven days and fifteen to twenty-one days for the parameters evaluated. In these phases, the addition of cellulase did not interfere with the enzymatic activity or protein levels in the liver. Enzyme measurement is useful for detecting recent or initial damage in birds rather than assessing normal organ function. Changes in enzyme activity (increases or decreases) can help in diagnosing bird health.

The mean concentrations of calcium, phosphorus, protein, chlorides, alkaline phosphatase, potassium, and calcium–phosphorus ratio in the blood serum of the birds in the pre-initial and initial phases are presented in Table 9.

There was a significant difference in the serum concentrations of calcium, phosphorus, chloride, and protein from one to seven days. For the period from eight to fourteen days, there was no significant difference except for alkaline phosphatase; however, there was a significant difference in electrolytes, the calcium–phosphorus ratio, and protein levels. For the parameters of calcium, chloride, protein, and potassium, there was a significant difference at 21 days of age.

The diet between Ca:P is very important for maintaining normal functions in birds. In feed, a Ca:P ratio of 2:1 is considered adequate; however, the diagnostic value of serum P in birds is not consistent, and the measurement of this mineral is seldom used for diagnosing a clinical condition [16].

The calcium/phosphorus ratio did not differ significantly between one and seven days and from fifteen to twenty-one days among the treatments, and in the period between eight and fourteen days, the ratio was significantly lower when the enzyme was not added to the feed. Due to the higher averages of calcium and phosphorus, there was an imbalance in the calcium/phosphorus ratio in the treatment without enzyme addition.

## 4. Discussion

The use of enzymes, specifically amylase, and cellulases, in feeds formulated with corn and soy affects the performance of broiler chickens. This is a result of the effect on feed consumption, leading to a significant weight gain despite reduced consumption compared to feeds without enzymes [17].

Contradictory results to this study were reported in reference [18]. They studied the performance of broiler chickens fed diets containing sorghum or millet with the addition of an enzyme complex from one to seven days and found no significant effect.

The birds’ digestive systems are anatomically complete after hatching, but their functional capacities for digestion and absorption are still immature. This may explain the increased feed intake when a higher amount of enzyme was added. When chicks hatch, they undergo a metabolic and physiological transition due to the switch from yolk sac feeding to exogenous feeding [19,20,21].

The multienzyme complex improves the performance of broiler chickens, with an increase in weight gain and better feed conversion due to being formed by amylase, cellulase, protease, and xylanase: enzymes that degrade the cellular material of the grains, releasing more nutrients into the intestinal lumen. This results in a greater absorption, thus improving performance [22].

In a study working with an enzyme complex composed of carbohydrases (amylase, protease, cellulase) in pelleted diets for male and female broiler chickens, it was concluded that, in the initial phase, the inclusion of 0.03% and 0.04% of the enzyme complex (amylase and cellulase) and the combination of 0.04% of the enzyme complex (amylase and cellulase) and 0.01% xylanase also resulted in better weight gain in males [23].

These authors, working with broiler diets based on corn and soybean meal, observed an increase in villus height and crypt depth, resulting in an increase in the absorption surface area, reflecting better broiler performance [24]. There was no statistical difference in weight gain and feed conversion between 15 and 21 days, but the addition of powdered cellulase at 500 g/t resulted in lower feed intake compared to the other treatments [25]. The addition of a multi-enzyme complex composed of cellulase, amylase, and protease to diets based on soybean meal, sorghum, soybean meal, and millet, resulted in no improvement in bird performance [26]. Due to the high specificity of enzymes in their catalytic reactions, products containing only one type of enzyme probably do not produce maximum benefits in poultry diets. This suggests that enzyme complexes may be more effective, as they act on a range of polysaccharides in grain cell walls (cellulose, arabinoxylans, β-glucans, xylans, pectins, etc.), leading to better diet utilization [27]. This may justify the lower feed intake with the addition of only cellulase from 15 to 21 days. These results suggest that adding enzymes to poultry diets can be targeted to specific periods, being more effective in the early life of birds when the gastrointestinal tract is not fully formed and cannot synthesize endogenous enzymes on a large scale.

When comparing the data from 1 to 17 days, when powdered cellulase at 1000 g/t was added, there was lower nitrogen consumption in the dry matter, a lower nitrogen balance, and consequently poorer retention. The treatments with basal diet, powdered cellulase at 500 g/t, and liquid cellulase did not differ significantly based on the nitrogen consumption in the dry matter, excretion, nitrogen balance, and retention. It is important to note that there are few studies based on the use of single exogenous enzymes, as many authors work with the addition of multi-enzyme complexes for formulating diets for broiler chickens. 

Nitrogen is one of the main pollutants in pig manure, which can be applied to poultry manure. The results of previous studies have demonstrated that in the pre-initial period from four to seven days, the addition of both powdered and liquid cellulase enzyme significantly reduced nitrogen excretion, improving nutrient utilization [28,29]. A similar result was found in reference [30]. The authors concluded that supplementing an enzymatic complex (xylanase, amylase, and cellulase) in the diet of broiler chickens from 1 to 21 days resulted in improved digestibility, suggesting an increase in the nutritional utilization of the diets. Similar results were found in the period from four to seven days. In this study, there was no significant difference in the nitrogen excreted in the dry matter and digestible nitrogen in the period from 14 to 17 days (Table 3).

Immediately after hatching, chicks utilize their limited body reserves to achieve rapid physical and functional development of the gastrointestinal tract in order to develop the capacity to digest food and assimilate nutrients [31]. Different feeding strategies can provide better nutrition for the embryo or chick after hatching, contributing to this metabolic and physiological transition and resulting in an increase in the birds’ performance [31,32]. The immaturity of the bird digestive system at this stage reduces their capacity to utilize nutrients. However, the digestibility results reported previously may suggest an increase in the nutritional utilization of diets and lower nitrogen excretion in the first few days of the birds’ life [33,34]. The authors of reference [35] found no differences in the weight of the liver, pancreas, proventriculus, gizzard, or the size and weight of the duodenum, jejunum, ileum, small intestine, and ceca of birds at 14 days of age with isoenergetic diets of wheat, corn and sorghum with the addition of exogenous enzymes.

High levels of non-starch polysaccharides increase both the size and relative weight of the duodenum, jejunum, ileum, and ceca. These increases in the intestines and the digestive system as a whole may be an adaptive response to the increased need for enzymes [36]. The use of powdered cellulase at a concentration of 1000 g/t showed the highest values for the relative weight of the small intestine, large intestine, and length of the gastrointestinal tract [37,38,39].

The protein levels in pre-starter diets were used to evaluate the biometry of the digestive system. The authors found no significant difference in the relative weight of the small intestine [32,40]. Another paper reported an increase in the surface area of intestine, followed by an increase in the villus length, which forced an increase in absorption and improvement in nutrient digestibility [41].

Particle size and food form also influence the size and weight of the gastrointestinal tract. One study evaluating the use of mash or pelleted diets made from medium (DGM = 1.54) or coarse (DGM = 1.69) particles found that birds at 21 days of age fed mash had relatively heavier gizzards and ceca and lighter jejunum compared to birds fed pellets [42].

Despite individual weight increases of the chicken, the percentage relationship of the digestive system weight to the bird weight decreases with age, and the temporal evolution of the digestive system weights observed in these experiments followed the expected trend with a reduction in their proportion with age of the chicken [43]. Another study demonstrated that the growth rate of the pancreas decreased linearly with bird age [44]. The maximum growth rate occurred on the seventh day of age, decreasing steadily until 28 days of age. The addition of exogenous amylase to the feed resulted in higher pancreatic amylase activities at 14 days of age. However, the authors concluded that the use of enzymes in broiler chicken diets did not have a persistent effect on pancreatic enzyme activity, which did not directly interfere with their production. This result is supported by these studies [45,46].

Pancreatic amylase, maltase, and sucrase reach maximum specific activity within three to four days. A study of four-day-old chicks demonstrated that starch digestion increased slightly between the 4th and 21st days of the bird’s life, from 82 to 89%, respectively [47,48].

It was observed that the maximum value of amylase in the pancreas occurs on the eighth day of life, while in the small intestine, the maximum value was found on the seventeenth day. These results may explain why there was no statistical difference between the treatments regarding amylase levels found in the 15 to 21-day period [49,50]. The results demonstrate that the addition of cellulase to the diet does not interfere with pancreatic amylase production or its absolute weight.

There is little information on the dosage of amylase in birds, and in the literature, there are few data available for discussion since the methodologies used vary due to the kits and equipment used [51]. Throughout the experimental period, there was no significant difference in the absolute weight of the liver with the addition of the cellulase enzyme. The results showed a progressive increase in the weight of the GIT as the bird’s age increased. This refers to the normal growth of viscera in rapidly growing birds.

The determination of the tissue enzymatic parameters of alkaline phosphatase (ALP), glutamate–oxaloacetate transaminase (GOT), glutamate–pyruvate transaminase (GPT), and liver protein levels are shown in Table 8. The production of alkaline phosphatase did not differ significantly during any of the phases evaluated. In the liver, it was not present in sufficient quantities to be significant, but an increase in its activity is usually reflective of a hepatic disorder, even though it is not a sensitive indicator [52,53,54]. Due to the osteoblastic activity that occurs during the growth phase, younger birds have higher values compared to adult animals. A decrease in its level was found in animals deficient in zinc, and it is believed that in cases of liver necrosis induced by aflatoxin B1, there is an enzymatic increase in ALP [53,55]. Decreased serum protein and albumin concentrations are reliable indicators of hepatotoxicity and acute or chronic inflammations in chickens and turkeys. In birds, the largest protein fraction (40–60%) is albumin, which is 100% synthesized in the liver. Therefore, its measurement can be a complementary aid in the diagnosis of liver diseases [56,57,58].

Low protein concentrations follow malnutrition, acute infections, and hemorrhage. The causes of hypoalbuminemia can be due to loss (via glomerular, enteropathies, skin lesions, and severe hemorrhages), decreased production (in cases of liver failure, poor diet, poor digestion, poor absorption), and decreased sequestration (avian ascites) [59,60,61].

Low GPT enzymatic activity in the liver can be demonstrated by the fact that many birds with hepatic disorders do not show changes in serum GPT levels. In some animals, GPT activity is so low that it cannot be detected by routinely used analyzers [53]. GOT activity exists in multiple tissues, with the liver and muscle being the main tissues. GOT is not specific for hepatocellular damage or muscle injury, but in psittacines, it always increases with hepatic damage of any etiology [62,63].

It is important to highlight the lack of reference intervals for many parameters. In avian clinics, it is common to use reference intervals based on the literature. However, in these studies, samples from a small number of animals were used, also were not properly characterized, and with no detailed descriptions of the methodologies used for laboratory tests. Most clinicians are aware of this issue and end up defining their own values based on individual experience [64,65]. However, due to the lack of updated results, discussing values of protein, FA, GOT, and GPT in the liver is difficult given the enormous variability of commercial kits and applied methodologies in birds.

Regarding the observation of the sanguine biochemical profile in the pre-initial and initial phases, it is known that serum nutrient profiles are presented in the literature for the different diets, strains, and analysis methodologies that are employed. Additionally, enzyme and mineral values in the serum differ among different studies, also due to the equipment used for the assays and the method of serum collection [66].

In birds, as in other species, minerals perform three types of functions:

- They are structural components of the GIT and tissues.
- They are constituents of the body fluids and tissues involved in maintaining osmotic pressure, acid–base balance, membrane permeability, and tissue irritability.
- They act as catalysts in enzymatic and hormonal systems and as integral or specific components of metalloenzymes’ structure [67,68].

Calcium is the most prevalent mineral in the body, forming part of bones and eggshells and playing an important role in many biochemical reactions [68]. The use of tested cellulases kept blood parameters the same regarding phosphorus levels, alkaline phosphatase, potassium, and the calcium/phosphorus ratio. Elevations in these elements can also occur in pathological conditions. Hypercalcemia (>11 mg/dL) has been associated with hypervitaminosis D3, osteolytic bone lesions secondary to neoplasms, and hyperalbuminemia [69]. On the other hand, hypocalcemia (<8 mg/dL) may be related to poor nutrition (vitamin D3 deficiency, excess dietary phosphorus), alkalosis, or hypoalbuminemia [62,70]. The results for the treatment containing the basal diet, even those that were significantly higher, were within the normal limits for birds.

During the period from one to seven days, the calcium levels were lower than the normal parameters for the birds when the cellulase enzyme was added to the feed. The introduction of enzymes into the chickens’ diet resulted in a decrease in bird weight and bone quality. Nevertheless, phytase supplementation restored the productivity of the chickens in the group treated with 600 FTU/kg BSP [71,72].

Regarding the calcium levels, they were only significantly different between 15 and 21 days, where the addition of cellulase powder at 1000 g/t was lower than in the other treatments. Ionic calcium values are not affected by albumin concentration, whereas total plasma calcium decreases with hypoalbuminemia and increases in hyperalbuminemia [62]. This explains the data found from one to seven days, where there was a decrease in protein and consequently the calcium levels in the blood for the enzyme-added treatments. The opposite occurred from 15 to 21 days, where with increased protein levels, there was an increase in calcium levels.

The macroelements calcium and phosphorus form the basis of skeletal formation, while sodium, chlorine, and potassium are distributed in higher concentrations in soft tissues, controlling acid–base balance [72,73,74]. 

The protein concentrations were significantly different throughout the analyzed period. When the cellulase enzyme was added to the feed, lower serum protein levels were observed, particularly at 15 to 21 days. The majority of the circulating proteins in the plasma are synthesized by the liver, except for immunoglobulins. The main functions of these molecules include maintaining blood volume through colloidal osmotic effects, participating in blood pH maintenance, hormone and drug transport, cellular coagulation, and catalyzing (enzymes) and regulating (hormones) biological processes [75]. Some proteins are also indispensable in inflammatory, immune, and tissue regeneration and repair processes, where they are called acute-phase proteins [59,76].

Due to the numerous functions of blood proteins, their elevation in serum can be due to many causes. This may explain the variations found in this study, where protein levels were higher from one to seven days and fifteen to twenty-one days and lower between eight and fourteen days when cellulase enzyme was not added to the feed. Decreases in protein concentrations have also been reported in situations of food restriction in broiler chickens, where hepatic protein synthesis was decreased by half [77]. Due to the high voracity and natural competition of birds for food, it is natural for some to go through periods without consuming food. This may justify the lower protein levels found at 15 to 21 days.

Male chicks fed with commercial feed based on corn and soy were evaluated for their amount of plasma protein using refractometry methodology for 52 weeks, obtaining protein concentration values of 3.30 g/dL in the first week. Similar concentrations were found in this study when the cellulase enzyme was used in all the evaluated periods [77,78].

The chloride concentrations were statistically different throughout the analyzed period. Potassium was only not statistically different at seven days but was also at 14 and 21 days. The broiler chickens fed with diets based on soybean meal and corn with various levels of potassium chloride were evaluated. The obtained average concentrations of chloride and potassium were 106.37 and 6.16 μmol/L, respectively. The averages identified for the chlorides in this study were lower than those mentioned previously for both chloride and potassium [79].

The degree of artificial changes (post-harvest) in the blood for the parameter of potassium seems to follow species-specific patterns [80]. Rapid processing of samples is essential to quantify this ion, as decreases of up to 60% in potassium concentrations in pigeon blood were observed after two hours of collection. In chickens, this decrease was 30%.

The alkaline phosphatase enzyme activity in the birds’ serum at 7, 14, and 21 days did not differ statistically. When evaluating alkaline phosphatase levels, it was observed that the enzyme levels in the serum were higher in the first week of the birds’ age, suggesting a greater metabolic demand and adaptation of the bird to the phase where there is no physiological maturity. It is noteworthy that these values increased, indicating that skeletal changes are related to bone remodeling in fast-growing broiler chicken strains [81].

The previously demonstrated alkaline phosphatase activity values for broiler chickens in the pre-initial phase fed commercial feed were 143.48 IU/L [82]. The authors evaluated whether continuous administration of probiotics to broiler chickens fed commercial feed would cause changes in serum enzyme levels of alkaline phosphatase and amylase [83]. They found that alkaline phosphatase showed activity peaks at seven days in control birds, showing no statistical difference from the treated birds (939.17 IU/L). Similar results were found during the evaluated phases with cellulase addition.

In previous work with amylase supplementation, the authors found alkaline phosphatase values that were not significant for 7 days of age (974.77 IU/L) and 21 days (966.90 IU/L). These results are similar to those of this study, except that enzymatic supplementation was performed using cellulase [51].

Hemolysis and prolonged contact with erythrocytes due to delayed plasma or serum separation can affect potassium, calcium, phosphorus concentrations, and serum alkaline phosphatase activity [69,72]. In birds, the 60 min deadline for sample centrifugation is not acceptable, as it is necessary for this to occur immediately after collection. With delay, the first change that occurs is a marked decrease in potassium values, which moves into the cells [6,84].

## 5. Conclusions

Supplementation of liquid and powdered cellulase enzyme in the feed at 21 days affected feed intake, digestibility, and blood electrolyte concentrations. The biometry of the gastrointestinal tract and analyses of pancreas and liver viscera were not altered, which may indicate normal bird development and the absence of toxicity.

## Figures and Tables

**Table 1:** Percentage and calculated composition of the experimental rations.

Ingredient (%)	1–7 Days	8–21 Days
Corn	57.58	60.04
Soybean meal	36.92	34.06
Soybean oil	1.29	2.17
Limestone	0.80	0.85
L-Lysine HCl	0.35	0.30
L-Threonine	0.13	0.10
Salt	0.44	0.42
Dicalcium phosphate	1.91	1.55
DL-Methionine	0.35	0.30
Vitamin premix ^1^	0.08	0.08
Mineral premix ^2^	0.10	0.10
Total	100.00	100.00
Calculated composition		
Crude protein, %	22.40	21.20
Metabolizable energy, Mcal/kg	2.96	3.05
Digestible lysine, %	1.32	1.21
Digestible methionine, %	0.66	0.59
Digestible met + cys, %	0.95	0.87
Calcium, %	0.92	0.84
Sodium, %	0.22	0.21
Available phosphorus, %	0.47	0.40
Digestible threonine, %	0.86	0.79
Digestible tryptophan, %	0.24	0.23

^1^ Vitamin A 5.3784 IU /Kg, vitamin D3 2.2744 IU/Kg, vitamin E 44.9894 IU/Kg, vitamin K 2239.37 mg/Kg, vitamin B1 1168.31 mg/Kg, vitamin B2 3585.60 mg/Kg, vitamin B6 1788.62 mg/Kg, vitamin B12 6723.00 mcg/Kg, folic acid 894.159 mg/Kg, nicotinic acid 22.402 g/Kg, pantothenic acid 8968.48 mg/Kg, biotin 89.64 mg/Kg, antioxidant 527, 7 mg/Kg, nicarbazin 50 mg/Kg, narasin 50.00 mg/Kg. ^2^ Mineral supplementation pre-starter feed (per kg of product): calcium 68.0000 g/kg, iron 30.0000 g/kg, zinc 40.0083 g/kg, selenium 225.0000 mg/kg, copper 75.0000 g/kg, manganese 45.0000 g/kg, iodine 500.0000 mg/kg, cobalt 3.0000 mg/kg magnesium 80.0000 mg/kg, zinc 17.9892 g/Kg.

**Table 2:** Ration consumption, weight gain, and feed conversion in pre-starter (1–7 days), (1–14 days) and starter (1–21 days) stage broilers chickens fed cellulase-supplemented rations.

Diet	1st to 7th Days			1st to 14th Days			1th to 21st Days		
	RC (g)	WG (g)	FC	RC (g)	WG (g)	FC	RC (g)	WG (g)	FC
**Basal diet (BD)**	124.85 ^b^	102.68	1.23	390.51	254.33 ^ab^	1.54 ^ab^	687.96 ^ab^	392.53	1.75
**BD + cellulase 500 g/t**	128.11 ^b^	101.01	1.24	375.88	275.75 ^a^	1.36 ^b^	654.80 ^b^	381.25	1.72
**BD + cellulase 1000 g/t**	142.16 ^a^	101.81	1.27	366.75	258.25 ^ab^	1.42 ^ab^	748.43 ^a^	405.75	1.84
**BD + cellulase 500 mL/t**	122.89 ^b^	98.98	1.40	388.76	238.03 ^b^	1.63 ^a^	727.13 ^a^	396.06	1.84
**CV (%)**	5.96	8.54	9.85	8.13	6.53	8.95	5.96	6.38	8.83
***p*** **-value**	0.00099	>0.05	0.0746	>0.05	0.0086	>0.05	0.0048	>0.05	>0.05
**SEM**	0.051	0.086	0.124	0.071	0.052	0.098	0.065	0.057	0.083

Coefficient of variation (CV); standard error of mean (SEM); ration consumption (RC); weight gain (WG); feed conversion (FC). a, b: significant at *p* < 0.01; a, b: significant at *p* < 0.05.

**Table 3:** Dry matter coefficient and nitrogen indices from 4 to 7-day-old and 14 to 17-day-old broiler chickens.

Diet	DDMC (%)	DMNI (g)	DMEN (g)	NB (g)	ND (%)	RETEN (%)
**4st to 7th days**						
**Basal diet (BD)**	36.63 ^b^	36.63 ^a^	5.75 ^a^	30.87 ^b^	84.01	27.76 ^c^
**BD + cellulase 500 g/t**	46.40 ^a^	30.92 ^b^	2.32 ^b^	41.74 ^a^	85.87	40.18 ^a^
**BD + cellulase 1000 g/t**	34.79 ^b^	35.35 ^ab^	1.44 ^c^	29.15 ^b^	83.67	27.91 ^c^
**BD + cellulase 500 mL/t**	38.39 ^b^	37.54 ^a^	2.30 ^b^	35.05 ^b^	83.42	31.72 ^b^
**CV (%)**	7.93	10.54	16.41	11.19	3.33	8.65
***p*** **-value**	<0.05	0.037	<0.05	<0.05	>0.05	<0.05
**SEM**	0.041	0.068	0.068	0.013	0.025	0.059
**14th to 17th days**						
**Basal diet (BD)**	66.42 ^a^	66.42	18.64	53.41 ^a^	90.18	47.63 ^a^
**BD + cellulase 500 g/t**	69.26 ^a^	69.94	18.18	55.60 ^a^	88.63	50.09 ^a^
**BD + cellulase 1000 g/t**	30.22 ^b^	67.48	18.24	13.05 ^b^	87.97	36.11 ^b^
**BD + cellulase 500 mL/t**	68.66 ^a^	67.89	17.85	55.13 ^a^	89.58	50.24 ^a^
**CV (%)**	3.87	7.38	8.04	6.24	2.54	8.65
***p*** **-value**	<0.05	0.527	>0.05	<0.05	0.364	<0.05
**SEM**	0.075	0.054	0.028	0.051	0.041	0.052

a, b: significant at *p* < 0.05. Means in the same column followed by different superscripted letters differ significantly according to the Tukey test (*p* < 0.05). BD: basal diet; BD + 500: basal diet + 500 mL/t cellulase; BD + 1000: basal diet + 1000 mL/t cellulase; coefficient of variation (CV); standard error of mean (SEM); dry matter digestibility coefficient (DDMC); dry matter nitrogen intake (DMNI); dry matter excreted nitrogen (DMEN); nitrogen balance (NB); nitrogen digestibility (ND); N retention (RETEN).

**Table 4:** Biometrics of the digestive system (relative weight) of 7, 14, and 21 day-old broiler chickens fed cellulase-supplemented rations.

Diet	GITM (cm)	GITW (%)	PROGI (%)	PANC (%)	LIV (%)	SI (%)	LI (%)
**1st to 7th days**							
**Basal diet (BD)**	52.05	7.25	8.08	0.51	4.22	5.89	1.36 ^b^
**BD + cellulase 500 g/t**	52.79	7.95	8.21	0.49	4.83	6.38	1.57 ^ab^
**BD + cellulase 1000 g/t**	57.99	8.21	8.55	0.55	4.72	6.51	1.70 ^a^
**BD + cellulase 500 mL/t**	57.35	9.92	8.03	0.53	4.67	6.43	1.49 ^ab^
**CV (%)**	11.55	12.45	14.16	12.73	10.75	13.91	8.01
***p*** **-value**	0.355	0.385	>0.05	0.226	0.184	>0.05	0.012
**SEM**	0.035	0.092	0.070	0.056	0.094	0.079	0.182
**8th to 14th days**							
**Basal diet (BD)**	26.70	5.42	4.53	0.34	2.69	4.38	1.13
**BD +Cellulase 500 g/t**	26.57	5.46	5.06	0.32	3.05	4.37	1.08
**BD + cellulase 1000 g/t**	30.13	5.76	5.41	0.36	2.99	4.70	1.05
**BD + cellulase 500 mL/t**	29.15	5.41	4.96	0.34	3.04	4.30	1.03
**CV (%)**	10.72	10.48	10.39	11.17	9.87	11.99	12.74
***p*** **-value**	0.195	0.381	0.058	>0.05	0.144	0.632	0.344
**SEM**	0.061	0.128	0.079	0.079	0.078	0.085	0.130
**15th to 21st days**							
**Basal diet (BD)**	15.82	4.36	3.76	0.26	2.47	3.56	0.80
**BD + cellulase 500 g/t**	16.18	4.27	3.87	0.25	2.38	3.40	0.86
**BD + cellulase 1000 g/t**	16.04	4.38	3.91	0.28	2.53	3.56	0.82
**BD + cellulase 500 mL/t**	16.27	4.31	3.93	0.29	2.55	3.51	0.84
**CV (%)**	10.38	9.00	9.86	10.82	9.83	11.63	7.12
***p*** **-value**	0.870	0.917	0.703	0.206	0.609	0.303	0.323
**SEM**	0.050	0.055	0.104	0.084	0.106	0.090	0.117

In the table, the letters a and b in the same column followed by different superscripted letters indicate that the values differ significantly according to the Tukey test (*p* < 0.05). a, b: significant at *p* < 0.01; a, b: significant at *p* < 0.05. BD: basal diet; BD + 500: basal diet + 500 mL/t cellulase; BD + 1000: basal diet + 1000 mL/t cellulase; coefficient of variation (CV); standard error of mean (SEM); gastrointestinal tract length (GITM); gastrointestinal tract weight (GITW); esophagus and crop weight (OESW); proventriculus and gizzard weight (PROGI); pancreas weight (PANC); liver weight (LIV); small intestine weight (SI); large intestine weight (LI).

**Table 5:** Averages of the absolute pancreas weight of 7, 14, and 21 day-old broiler chickens fed cellulase-supplemented rations.

Diet	Pancreas (g) 1st to 7th Days	Pancreas (g) 8th to 14th Days	Pancreas (g) 15th to 21st Days
**Basal diet (BD)**	0.88	1.53	2.44
**BD + cellulase 500 g/t**	0.80	1.58	2.41
**BD + cellulase 1000 g/t**	0.80	1.48	2.48
**BD + cellulase 500 mL/t**	0.90	1.48	2.35
**CV (%)**	6.22	5.66	6.33
***p*** **-value**	0.142	>0.055	>0.059
**SEM**	0.062	0.056	0.063

**Table 6:** Pancreatic amylase activity in the different evaluated phases.

Diet	Amylase (IU/L) 1st to 7th Days	Amylase (IU/L) 8th to 14th Days	Amylase (IU/L) 15th to 21st Days
**Basal diet (BD)**	794.38	793.36	780.28
**BD + cellulase 500 g/t**	798.64	799.6	795.43
**BD + cellulase 1000 g/t**	798.62	802.52	803.67
**BD + cellulase 500 mL/t**	799.52	775.94	801.08
**CV (%)**	2.31	1.53	1.31
***p*** **-value**	>0.05	>0.05	0.35
**SEM**	0.028	0.015	0.039

**Table 7:** Average absolute liver weight for the different treatments during the experimental period.

Average Absolute Liver (g)			
Diet	1st to 7th Days	8th to 14th Days	15th to 21st Days
**Basal diet (BD)**	6.20	12.01	22.26
**BD + cellulase 500 g/t**	6.38	12.91	23.85
**BD + cellulase 1000 g/t**	5.83	13.21	25.15
**BD + cellulase 500 mL/t**	5.83	13.30	25.13
**CV (%)**	8.41	9.59	12.08
***p*** **-value**	0.236	0.120	0.322
**SEM**	0.042	0.058	0.054

**Table 8:** Concentrations of protein (Prot) and enzymes alkaline phosphatase (ALP), glutamate–oxaloacetate transaminase (GOT), and glutamate–pyruvate transaminase (GPT) in the livers from 1 to 21 days.

	1st to 7th Days			
Diet	Prot (mg/dL)	ALP (IU/L)	GOT (IU/L)	GPT (IU/L)
**Basal diet (BD)**	2.07	212.13	272.04	31.96
**BD + cellulase 500 g/t**	2.32	206.58	262.39	27.99
**BD + cellulase 1000 g/t**	2.12	205.78	270.68	29.87
**BD + cellulase 500 mL/t**	2.30	211.58	266.62	29.16
**CV (%)**	8.84	5.67	11.78	10.61
***p*** **-value**	0.205	>0.05	>0.05	>0.05
**SEM**	0.047	0.015	0.098	0.042
	**8th to 14th days**			
**Rations**	**Prot (mg/dL)**	**FA (IU/L)**	**GOT (IU/L)**	**GPT (IU/L)**
**Basal diet (BD)**	2.62 ^a^	220.06	290.98	25.65 ^b^
**BD + cellulase 500 g/t**	1.86 ^b^	208.26	280.37	24.81 ^ab^
**BD + cellulase 1000 g/t**	1.61 ^b^	215.22	281.81	26.63 ^a^
**BD + cellulase 500 mL/t**	1.76 ^b^	210.63	284.09	26.68 ^a^
**CV (%)**	9.05	9.34	9.07	7.89
***p*** **-value**	<0.05	>0.05	0.197	0.357
**SEM**	0.029	0.042	0.031	0.034
	**15th to 21st days**			
**Diet**	**Prot (mg/dL)**	**FA (IU/L)**	**GOT (IU/L)**	**GPT (IU/L)**
**Basal diet (BD)**	2.14	209.00	262.04	31.09
**BD + cellulase 500 g/t**	2.22	206.58	262.39	28.74
**BD + cellulase 1000 g/t**	2.12	205.78	265.68	29.98
**BD + cellulase 500 mL/t**	2.16	207.88	266.62	29.80
**CV (%)**	5.29	5.52	11.78	10.39
***p*** **-value**	>0.05	>0.05	>0.05	>0.05
**SEM**	0.022	0.068	0.097	0.033

In the table, the letters a and b in the same column followed by different superscripted letters indicate that the values differ significantly according to the Tukey test (*p* < 0.05). a, b: significant at *p* < 0.01; a, b: significant at *p* < 0.05.

**Table 9:** Serum levels of minerals during the experimental period.

	1st to 7th Days						
Diet	Calcium (mg/dL)	Phosphorus (mmol/L)	Ratio of Ca:P	Chloride (mmol/L)	Potassium (mmol/L)	Protein (mg/dL)	Alkaline Phosphatase (IU/L)
**Basal diet (BD)**	10.85 ^a^	5.57 ^a^	1.95	81.92 ^b^	6.55	3.55 ^a^	213.13
**BD + cellulase 500 g/t**	7.15 ^b^	3.51 ^b^	2.01	100.27 ^a^	6.09	2.11 ^b^	229.09
**BD + cellulase 1000 g/t**	7.24 ^b^	3.36 ^b^	2.15	90.77 ^ab^	6.35	2.06 ^b^	218.80
**BD + cellulase 500 mL/t**	6.81 ^b^	3.12 ^b^	2.19	99.68 ^a^	6.20	2.26 ^b^	228.38
**CV (%)**	8.12	7.24	8.49	8.47	8.49	7.61	7.34
***p*** **-value**	<0.05	<0.05	0.18	<0.05	>0.05	<0.05	0.179
**SEM**	0.023	0.029	0.054	0.092	0.031	0.028	0.034
	**8th to 14th days**						
**Basal diet (BD)**	9.36 ^b^	5.50 ^a^	1.70 ^a^	86.57 ^a^	7.71 ^a^	2.54 ^b^	260.08
**BD + cellulase 500 g/t**	9.85 ^a^	5.05 ^b^	1.95 ^b^	69.39 ^b^	6.41 ^b^	2.88 ^a^	261.19
**BD + cellulase 1000 g/t**	9.82 ^a^	5.07 ^b^	1.94 ^b^	67.19 ^b^	6.49 ^b^	2.89 ^a^	264.42
**BD + cellulase 500 mL/t**	9.55 ^ab^	5.03 ^b^	1.90 ^b^	69.93 ^b^	6.13 ^b^	2.84 ^ab^	259.1
**CV (%)**	9.16	5.05	6.25	8.71	6.82	5.97	5.88
***p*** **-value**	0.0015	0.013	0.002	<0.05	>0.06	0.019	>0.05
**SEM**	0.024	0.043	0.062	0.122	0.104	0.059	0.088
	**15th to 21st days**						
**Basal diet (BD)**	10.97 ^a^	5.53	2.00	74.47 ^b^	8.19 ^a^	3.22 ^a^	206.57
**BD + cellulase 500 g/t**	10.53 ^a^	5.23	2.02	90.21 ^a^	7.18 ^b^	2.56 ^b^	208.82
**BD + cellulase 1000 g/t**	9.85 ^b^	5.17	1.90	90.72 ^a^	7.27 ^b^	2.65 ^b^	208.50
**BD + cellulase 500 mL/t**	10.98 ^a^	5.59	1.96	94.34 ^a^	7.10 ^b^	2.52 ^b^	203.43
**CV (%)**	5.05	5.91	4.80	9.41	6.44	9.79	8.98
***p*** **-value**	<0.05	0.263	>0.05	<0.05	<0.05	<0.05	>0.05
**SEM**	0.052	0.039	0.026	0.101	0.068	0.119	0.019

In the table, the letters a and b in the same column followed by different superscripted letters indicate that the values differ significantly according to the Tukey test (*p* < 0.05). a, b: significant at *p* < 0.01; a, b: significant at *p* < 0.05.

## Data Availability

The data are contained within the article.
